# INTEGRAL ROLE OF SOCIAL WORKERS WITHIN A COMPREHENSIVE GERIATRIC ASSESSMENT

**DOI:** 10.1093/geroni/igad104.2257

**Published:** 2023-12-21

**Authors:** Ashley Schwartzkopf, Jennifer Myers, Cathy Schubert, Lauren Penney, Teresa Damush, Aliana Preddie, Dawn Bravata

**Affiliations:** Richard L Roudebush VA Medical Center, Indianapolis, Indiana, United States; Richard L Roudebush VA Medical Center, Indianapolis, Indiana, United States; Richard L Roudebush VA Medical Center, Indianapolis, Indiana, United States; National Institutes of Health, Bethesda, Maryland, United States; Richard L Roudebush VA Medical Center, Indianapolis, Indiana, United States; Richard L Roudebush VA Medical Center, Indianapolis, Indiana, United States; Richard L Roudebush VA Medical Center, Indianapolis, Indiana, United States

## Abstract

Comprehensive geriatric assessment (CGA) programs are expanding to support the wellbeing of community-dwelling older adults. Although a variety of CGA models have been described, the specific contributions of social work in CGA programs has not been rigorously evaluated. We examined the role of social work within a well-established, interdisciplinary, home-based geriatric program at one large, midwestern VA Medical Center. Semi-structured interviews were conducted with the VA-Geriatric Resources for Assessment and Care of Elders (GRACE) team to understand their perspective on social work expertise within a geriatric interdisciplinary team. Nine VA-GRACE team members were interviewed: 1 geriatrician, 1 pharmacist, 4 advanced practice nurses, and 3 social workers. VA-GRACE social workers participated in home-based CGAs by identifying psychosocial barriers that affected patients’ medical care and quality of life, and engaging in ongoing case management. VA-GRACE team members valued the social workers problem-solving skills which were critical to ensuring that: patients received needed home services; caregiver needs were met; home safety and equipment issues were addressed; and patients received appropriate cognitive services. The VA-GRACE team members valued social workers for their perceived positive work ethic, as well as their critical knowledgebase which they apply to training new staff, supporting nurses (especially during periods of staff shortage), promoting patient enrollment, and developing and implementing cohesive care plans. In conclusion, these results support the integral role of social work in an interdisciplinary team providing comprehensive geriatric assessments and highlight unique contributions that social workers make to promote the health and wellbeing of older adults.

